# Tape Casting of LLZO Ceramic Separators: An Overview of Challenges, Optimization Strategies, and Paths to Industrial Implementation

**DOI:** 10.1002/cssc.202502488

**Published:** 2026-04-05

**Authors:** Kaouther Touidjine, Ruijie Ye, Melanie Finsterbusch‐Rosen, Artur Lang, Martin Finsterbusch, Dina Fattakhova‐Rohlfing

**Affiliations:** ^1^ Institute of Energy Materials and Devices IMD‐2: Materials Synthesis and Processing Forschungszentrum Jülich GmbH Jülich Germany; ^2^ Faculty of Engineering and Center for Nanointegration Duisburg‐Essen CENIDE University Duisburg‐Essen Duisburg Germany; ^3^ Helmholtz Institute Münster: Ionics in Energy Storage (IMD‐4) Forschungszentrum Jülich GmbH Münster Germany

**Keywords:** battery separator, LLZO, solid‐state battery, solid‐state electrolyte, tape casting

## Abstract

Tape casting is a cost‐effective process for the scalable and continuous production of thin layers from ceramic powders. In recent years, interest in the use of tape casting for the production of solid‐state battery components such as ceramic separators, ceramic cathodes, or complete cells has grown significantly. This upswing has been particularly pronounced for ceramic battery components based on the garnet‐type Li_7_La_3_Zr_2_O_12_ (LLZO) solid electrolyte, as it exhibits the highest chemical stability toward Li metal anodes, while also possessing high overall ionic conductivity and being processable under ambient conditions. Since the separator has a decisive influence on the battery performance, strict control of the tape casting process at every stage, from slurry formulation and sintering to posttreatment and cell integration, is crucial for the reproducible manufacture of LLZO battery components with satisfactory performance. This perspective provides a systematic analysis of the challenges of the LLZO tape casting process and examines their impact on morphology, phase stability, and electrochemical performance, which often lead to nonreproducible results in various studies. In addition to presenting state‐of‐the‐art solutions to these interrelated problems, we offer our perspective and propose innovative approaches to address concerns about scalability and reproducibility. By identifying critical gaps and opportunities for future research and development, this overview aims to facilitate understanding of the real challenges in the industrialization of LLZO separators and ultimately contributes to the realization of commercially viable all‐solid‐state batteries.

## 1 Introduction

Global demand for rechargeable batteries is growing rapidly, driven by electrification in transportation, consumer electronics, medical devices, and industrial tools. According to the IEA, the annual demand for batteries for electric vehicles (EVs) alone is expected to exceed 3 TWh by 2030 [1], while additional growth is expected from safety‐critical and high‐performance applications such as hearing aids, power tools, and aerospace systems.

Although Li‐ion batteries (LIBs) are reliably powering portable electronics and EVs for decades, they are gradually reaching their intrinsic limits in terms of capacity (typically < 300 Wh/kg) and range [2, 3], with most EVs currently offering a range of 300–500 km per charge [4]. Beyond performance, safety remains a critical challenge for a wide range of battery applications, as the number of battery‐related incidents increases with the growing popularity of EVs, electric scooters, and e‐bikes. The use of flammable liquid electrolytes poses risks such as leakage, gas evolution, and thermal runaway, which have been reported for EVs [5]. Due to the direct correlation between the battery energy density and accident severity [6], as well as the generation of highly toxic by‐products in battery fires, such as HF, HCl, and SO_2_, which can contaminate air, groundwater, and soil [7], significant efforts are being made to improve battery safety.

The limitations of energy density and the safety risks of current LIBs have increased industrial interest in solid‐state batteries (SSBs), which are widely regarded as an important next‐generation technology. Major automotive manufacturers such as Toyota, Volkswagen, BMW, and Hyundai have announced SSB development programs, with commercialization targeted for 2030 [8]. In addition to automotive programs, the development of SSBs is also gaining importance in a variety of other industries. Electronics suppliers such as TDK [9] or Samsung SDI [10, 11] have announced solid‐state prototypes for wearables, hearing aids, smartphones, laptops, and other small devices, which have a reported energy density significantly higher than current lithium‐ion cells and offer improved safety for personal electronic devices. SSBs currently account for about 0.5% of the global battery market, which is dominated by polymer‐based batteries. A recent study by Fraunhofer institute [12] predicts an increase to about 1% by 2035, driven by the successful commercialization of oxide‐ and sulfide‐based battery systems.

SSBs use a solid electrolyte that enables the safe use of lithium metal, which offers the highest theoretical capacity (3860 mA h g^−1^) [13] and eliminates the intercalation limitations that restrict fast charging in graphite anodes in traditional LIBs [14]. By replacing the flammable liquid electrolyte with a chemically stable, nonflammable, and mechanically rigid solid medium, solid‐state electrolytes can suppress the penetration of dendritic lithium, which typically leads to short circuits and thermal runaway in conventional LIBs [15]. The combination of lithium metal compatibility and increased safety directly contributes to higher energy density at the cell level, enables denser component packing, and reduces self‐discharge [16].

Several classes of solid electrolytes have been developed, including perovskites, garnets, NASICON‐type conductors, sulfides, argyrodites, LIPON, and halides. Among these, sulfide‐based and garnet‐type oxide electrolytes have emerged as particularly promising candidates for high‐energy SSBs [17, 18]. Sulfide electrolytes such as Li_10_GeP_2_S_12_ exhibit conductivities of up to ∼10^−2^ S cm^−1^ [19], but their low chemical stability and the formation of toxic H_2_S upon contact with ambient atmosphere pose significant challenges in manufacturing, while the need for high pressures during operation makes scalability to a larger cell format difficult [20]. In contrast, the garnet‐type Li_7_La_3_Zr_2_O_12_ (LLZO) combines high ionic conductivity of up to 2 × 10^−3^ S cm^−1^ [21], nonflammability, and compatibility with lithium metal [22], establishing itself as a promising separator material for lithium‐metal and zero‐excess lithium cell configurations.

However, in order to take advantage of these beneficial properties, mechanically robust separators with a thickness comparable to that of separators in conventional LIBs must be produced on a large scale [23, 24]. Since LLZO is typically available as a ceramic powder, it requires shaping into green tapes or pellets followed by high‐temperature sintering to obtain dense, mechanically stable components. Thick pellets (∼500 µm), which are commonly used in research [25, 26, 27], are unsuitable for practical cells because they reduce energy density, increase internal resistance, and offer limited scalability. *Rupp* et al. [28] showed that competitive energy and power densities in oxide‐based SSBs critically depend on the production of thin, homogeneous LLZO films with high ionic conductivity. To achieve practical performance, these films typically need to be less than 20 µm thick and have an ionic conductivity of at least 1 mS cm^−1^ [29]. In line with this, several authors [29, 30] have identified wet‐coating processes such as tape casting as one of the most realistic manufacturing methods for large‐scale oxide‐based SSBs, projecting that the cell‐level costs for garnet systems could fall below 150 USD kWh^−1^ if the production costs for solid electrolyte are reduced sufficiently.

Tape casting is an established industrial process that offers numerous advantages in the production of free‐standing ceramic layers. It does not require complex infrastructure, is scalable, can be performed continuously, and is relatively inexpensive [31, 32]. It offers a high degree of flexibility in controlling the thickness, microstructure, and properties of the resulting layers, mainly achieved by adjusting the slurry formulations, casting parameters, and sintering regimes, and is suitable for the production of complex multilayer component architectures with different microstructures [32]. In particular, tape casting enables the easy production of multilayer and functionally graded structures at the green‐body stage, which are difficult to realize with batch‐type pressing or spark plasma sintering (SPS), especially for large areas and high throughput.

In a typical tape casting process, ceramic powder is mechanically dispersed in a solvent with a dispersant to stabilize the particles. Binders and plasticizers are added to create a viscoelastic slurry that can be cast onto a stationary or moving substrate to form a cohesive green tape. After drying, the green tape can be removed, shaped, and sintered into mechanically stable thin ceramic components. The thickness, microstructure, and mechanical properties of the final layers critically depend on the slurry composition and rheological properties [33, 34].

Although research into the application of tape casting process for the production of LLZO separators is relatively new, numerous studies have already confirmed the feasibility of this technique for the fabrication of high‐performance LLZO separators with high ionic conductivity and operating at high capacity with Li metal anodes [35]. However, despite the progress made, the fabrication of tape‐cast LLZO separators with satisfactory properties remains a challenge, particularly in terms of process reproducibility and the reliable performance of the final separators in SSBs. The main limitation is not the tape casting process itself, which is applicable to LLZO, but the intrinsic chemical reactivity of the LLZO structure, which differs significantly from that of the metal oxides used in the ceramic industry [29, 36, 37, 38, 39].

The biggest challenges are related to the presence of highly mobile Li ions in the LLZO structure, which readily undergo proton exchange in humid air and protic solvents [40, 41, 42, 43]. Lithium‐proton exchange during various stages of the tape casting process leads to undesirable phase transformations of LLZO and the formation of insulating Li_2_CO_3_ at the surface [44], which impairs the conductivity and structural integrity of the final separators. The high mobility of Li‐metal ions also poses a problem during the sintering process, as they tend to leave the structure at elevated temperatures (known as lithium loss) and must be compensated for by adding excess lithium before sintering [38, 45]. The problematic formation of Li_2_CO_3_ and the associated Li loss from the crystal structure, combined with the evaporation of volatile Li species during sintering, strongly influence the microstructure [46], chemical composition [47], and grain boundary properties of the resulting separators [48], ultimately compromising their performance in batteries.

The successful production of LLZO separators for SSBs using tape casting requires not only optimization of the manufacturing process, but also an understanding of the critical physical and chemical phenomena during the various fabrication steps and their role in controlling the properties of the final components [49]. Therefore, this perspective article aims to provide a systematic overview of the challenges at the mechanistic level that must be considered when manufacturing thin LLZO ceramic separators using tape casting. Starting with the tape‐casting slurry, through the sintering conditions to the postsintering treatments, the influence of processing parameters and lithium‐proton exchange on the mechanical and electrochemical properties of the final separator is elucidated. The following sections therefore present the most successful strategies for manufacturing high‐performance LLZO‐based separators using tape casting to further advance efforts toward their industrial implementation in next‐generation batteries.

## 2 Manufacturing Ceramic LLZO Separators via Tape Casting

**FIGURE 1:**
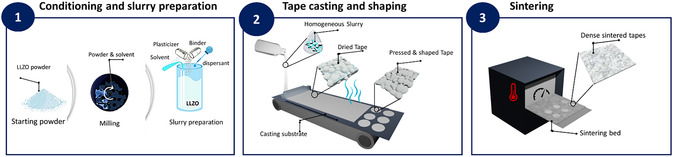
Illustration of a standard tape casting procedure for manufacturing LLZO separators.

The tape casting process for manufacturing LLZO separators comprises three main steps, which are shown schematically in Figure 1. In Step 1 (conditioning and slurry preparation), the LLZO powder is conditioned by milling to achieve an optimized particle size and particle size distribution. A slurry is then prepared by dispersing the milled LLZO powder in a suitable solvent together with organic additives. In the subsequent Step 2 (tape casting and shaping), the resulting slurry is cast onto a selected substrate (usually a polymer film) and dried to produce a flexible green tape. The green tape is then removed from the substrate and cut or punched to obtain separators of the desired size and shape. These cutouts are then transferred to a heat‐resistant substrate and sintered in Step 3 (sintering) to obtain the final rigid thin ceramic layer. For battery manufacturing, the integration of such layers, for example, as separator, requires good mechanical stability and flatness.

This section focuses mainly on the first two steps, in particular on the optimization of the slurry formulation and its impact on the casting step. Although the integration of the separators into the cell is not explicitly addressed, the important properties of the final separators (such as ionic conductivity, microstructure, flatness, and performance with Li anodes) are discussed as important quality criteria of the resulting LLZO separators. The specific challenges of the sintering process are addressed separately in Section 4.

The preparation of the slurry is a crucial step, as it directly affects the properties of the green tapes and the final sintered separators. Factors such as density, porosity, and mechanical and electrochemical stability are closely related to the composition of the slurry and its processing. Before the LLZO powder is processed into a slurry, it is usually subjected to pretreatment (conditioning) to reduce the particle size, typically from the micrometer to the submicrometer range. This is achieved by milling the starting powders either in a dry milling process or in a wet milling in a suitable solvent [50, 51]. Milled LLZO particles are then dispersed in a solvent together with binders, dispersants, and plasticizers. These additives control particle–particle interactions, stabilize the suspension, prevent sedimentation, and define the rheological properties of the slurry required for tape casting. After drying, the binders form a polymeric matrix that provides the mechanical integrity and flexibility of the green tape. In accordance with the classical tape casting process described by Mistler [52], the production of a well‐dispersed, homogeneous slurry is therefore one of the most critical prerequisites for obtaining dimensionally stable, defect‐free green tapes and, ultimately, high‐quality sintered LLZO separators. Table 1 provides an overview of various LLZO slurry formulations available in the literature and highlights the different solvent–binder systems, dispersants, plasticizers, and additives that have been used to date for tape‐cast LLZO separators.

**TABLE 1:** Overview of reported slurry recipes for tape casting LLZO separators and the important properties (ionic conductivity and relative density) of the sintered tapes.

Solvent	Binder	Dispersant	Plasticizer	LLZO: structure dopant	Sinter aid	Relative density of tapes after sintering, %	Conductivity of tapes after sintering, S cm^−1^	Ref.
Ethanol	PVB	Menhaden fish oil	Polyethylene glycol	LLZO:Al	ZnO		0.8·10^−4^	[53]
Ethanol, butanol	PVB	Alkylammonium salt of a copolymer	PEG	LLZO:Al, Ta		92.8	3.9·10^−4^	[37]
Ethanol, butanol	PVB	Alkylammonium salt of a copolymer	PEG	LLZO:Al, Ta	Li_2_CO_3_	95	1.8·10^−4^	[46]
Ethanol, toluene	Ethyl‐cellulose	Menhaden fish oil	Dibutyl phthalate, polyethylene glycol	LLZO:Nb	Li_3_BO_3_	90.8	2.8·10^−4^	[54]
Ethanol, acetone	PVB		Benzyl butyl phthalate	LLZO:Al	MgO	92.3	2.25·10^−4^	[55]
Ethanol, butyl acetate	Polyacrylic resin		Polyacrylic resin	LLZO:Ta	Li_2_O	99	5.2·10^−4^	[56]
Ethanol, acetone	PVB	PAA	Benzyl butyl phthalate	LLZO:Al		94	2·10^−4^	[57]
Water	Methyl‐cellulose		PEG, glycol	LLZO:Al, Ta		90	1.5·10^−4^	[58]
Water	Acrylic emulsion, xanthan gum	Ammoniumpolymethylacrylate		LLZO:Al		95	5·10^−4^	[59]
Water, ethanol	Methyl‐cellulose	Dispex Ultra PA4560	PEG, glycerol	LLZO:Al	MgO	91	2.3·10^−4^	[55]
Toluene	MSB1−13	DS002 (phosphate ester)		LLZO:Al	MgO	87.9	1.7·10^−4^	[55]
Toluene, isopropanol	PVB	Menhaden fish oil	PAG	LLZO:Nb		Porous bilayer	5.35·10^−4^	[60]
Toluene, isopropanol	PVB	Menhaden fish oil	Benzyl butyl phthalate	LLZO:Ca, Nb		Porous bilayer	2.2·10^−4^	[61, 62]
Toluene, isopropanol	PVB	Menhaden fish oil	Benzyl butyl phthalate	LLZO:Nb, Ca		ca. 99 (dense)		[63]
Toluene, isopropanol	PVB	Menhaden fish oil	Benzyl butyl phthalate	LLZO:Al		>90		[63]
Ethylene glycol monoethyl ether	PVB		Glycerol trioleate	LLZO:Ta		Porous		[64]

When selecting organic additives and solvents, chemical compatibility with LLZO must be ensured in order to avoid undesirable reactions that could impair its stability. The solvent must either promote the effective formation of a stable organic matrix by dissolving the binder and other organic components or form a stable dispersion while maintaining an optimal balance between chemical stability with the LLZO, that is, not causing protonation or Li_2_CO_3_ formation. At the same time, undesired side reactions such as polymerization must be avoided in order to ensure the stability and processability of the slurry. Sintering aids are often added to the slurry to improve densification during the heat treatment step. Therefore, their stability within the mixture must also be taken into account. After casting and drying the slurry, the green tapes must have sufficient cohesion and flexibility to withstand subsequent processing steps such as lamination and shaping. Final densification is achieved during heat treatment, during which the organic components are removed (binder burnout) and the LLZO microstructure is solidified.

Numerous slurry formulations have been developed to successfully cast LLZO into thin films, as illustrated in Table 1. The selection of binders and solvents is particularly critical. Commonly used binders such as polyvinyl butyral (PVB) and ethyl cellulose are preferred due to their solubility in solvents such as ethanol and toluene**,** which are generally compatible with LLZO and can also burn out at relatively low temperatures during sintering, facilitating the densification of ceramic tapes. Polyethylene glycol is typically used as a plasticizer, while dispersants are either polymer‐ or surfactant‐based. While the choice of solvents and binders is primarily determined by technical factors such as slurry rheology, green tape flexibility, and final performance, safety and scalability considerations impose additional constraints. Water‐based systems are highly desirable for industrial applications due to their safety, environmental compatibility, and ease of handling. However, water is not always ideal for casting LLZO tapes, as it can promote the protonation LLZO**,** leading to undesirable lithium‐proton exchange and possible degradation. In addition, aqueous slurries often suffer from poor wetting due to their higher surface tension. Being polar, they can cause agglomeration through proton bonding, thus impairing slurry flow. On the other hand, organic solvents such as toluene, ethanol, and acetone offer better rheological control but pose challenges for large‐scale production due to their flammability, toxicity, and environmental concerns**.** Binder systems such as PVB, which require phthalate‐based plasticizers**,** raise additional safety and regulatory issues. These trade‐offs must be carefully weighted to optimize both the castability and performance of LLZO slurries while ensuring industrial viability. For example, *Johnson* et al*.* highlighted the technical challenge of finding the optimum slurry formulation by screening different binders and solvents (Table 2 [55]).

**TABLE 2:** Guiding criteria for selecting possible slurry formulations for tape casting LLZO layers (adapted from [55]).

Binder system	Solvent	LLZO compatibility	Slurry foaming	Ease of carrier (support) release	Mechanical properties of green tape	Oxide‐only density of laminates, %
PVA	Aqueous	Gels	—	—	—	—
PII–WB4101	Aqueous	Gels	—	—	—	—
Methylcellulose	Aqueous	Yes	Yes, controllable	Controllable dewetting	Good	40.2
PVB	Ethanol/acetone	Yes	No	Poor	Brittle	44.3
Ethyl‐cellulose	Toluene/ethanol	Yes	Minor	Good	Brittle	43.9
PII–MSB1‐13	Toluene/xylene	Yes	No	Good	Very good	41.7

Defining a single “best” slurry formulation is inherently difficult, as the selection process requires a compromise between technical feasibility, safety considerations, and reproducibility on a large scale. While hazardous solvents and additives can be carefully handled under controlled conditions on a laboratory scale, industrial‐scale production is subject to stricter safety regulations and requires consistent process stability. The transition from feasibility at laboratory scale to commercial viability requires a balance between rheological performance, LLZO compatibility, environmental impact, and handling risks. To provide a clear comparative perspective, we have created a table (Table 3) that evaluates key slurry components based on their ability to meet the most important criteria for LLZO tape casting. This classification serves as a practical guide for researchers and engineers in selecting optimal binder–solvent–dispersant systems that meet both scientific requirements and industrial constraints.

**TABLE 3:** Estimation of the suitability of various binders, solvents, and dispersants for formulating slurries for LLZO tape casting based on the rheological properties of the slurry, process safety, and performance of final LLZO separators in symmetrical Li/LLZO/Li cells.

	Component	Good for rheology	Toxicity	Safety for large‐scale production	Good for performance of LLZO separators
Binder	PVA (water‐soluble, gel‐forming)	No	No	Yes	No
	PVB (solvent‐based, flexible)	Yes	Yes	No	Yes
	Ethylcellulose (thermoplastic, strong adhesion)	Yes	Yes	No	Yes
Solvent	Water	No	No	Yes	No
	Ethanol (moderate evaporation, safe)	Yes	Moderate	Moderate	Moderate
	Isopropanol (slower evaporation, safer)	Yes	Moderate	Moderate	Moderate
	Toluene (low surface tension)	Yes	Yes	No	Yes
	Acetone (fast evaporation)	Moderate	Yes	No	Yes
Dispersant	Menhaden fish oil	Moderate	No	Yes	Yes
	Dispex Ultra PA4560 (polymeric, strong stabilization)	Yes	No	Yes	Yes
	Polyacrylic acid (electrostatic stabilization)	Moderate	No	Yes	Moderate
	Polyethylene glycol	Yes	No	Yes	Yes

### 2.1 Main Challenges During the Slurry Fabrication

The surface of LLZO powders is sensitive to water [40] (including moisture [41, 42, 43, 65]), carbon dioxide [66], and protic solvents [67]. This sensitivity is due to a lithium‐proton exchange (also called protonation), which is explained in more detail below. Although protonation plays a role in all LLZO manufacturing processes, it is particularly important in tape casting because there are a greater number of different processing steps in which the LLZO powder comes into contact with either solvents or the atmosphere. Therefore, it is essential to understand the mechanism of LLZO protonation in the context of tape casting in order to identify the parameters that enhance undesirable interactions and to develop strategies to counteract this effect. This is especially important as the use of environmentally friendly solvents and processing in air reduces the cost of manufacturing LLZO‐based components.

#### 2.1.1 Protonation Mechanism

**FIGURE 2:**
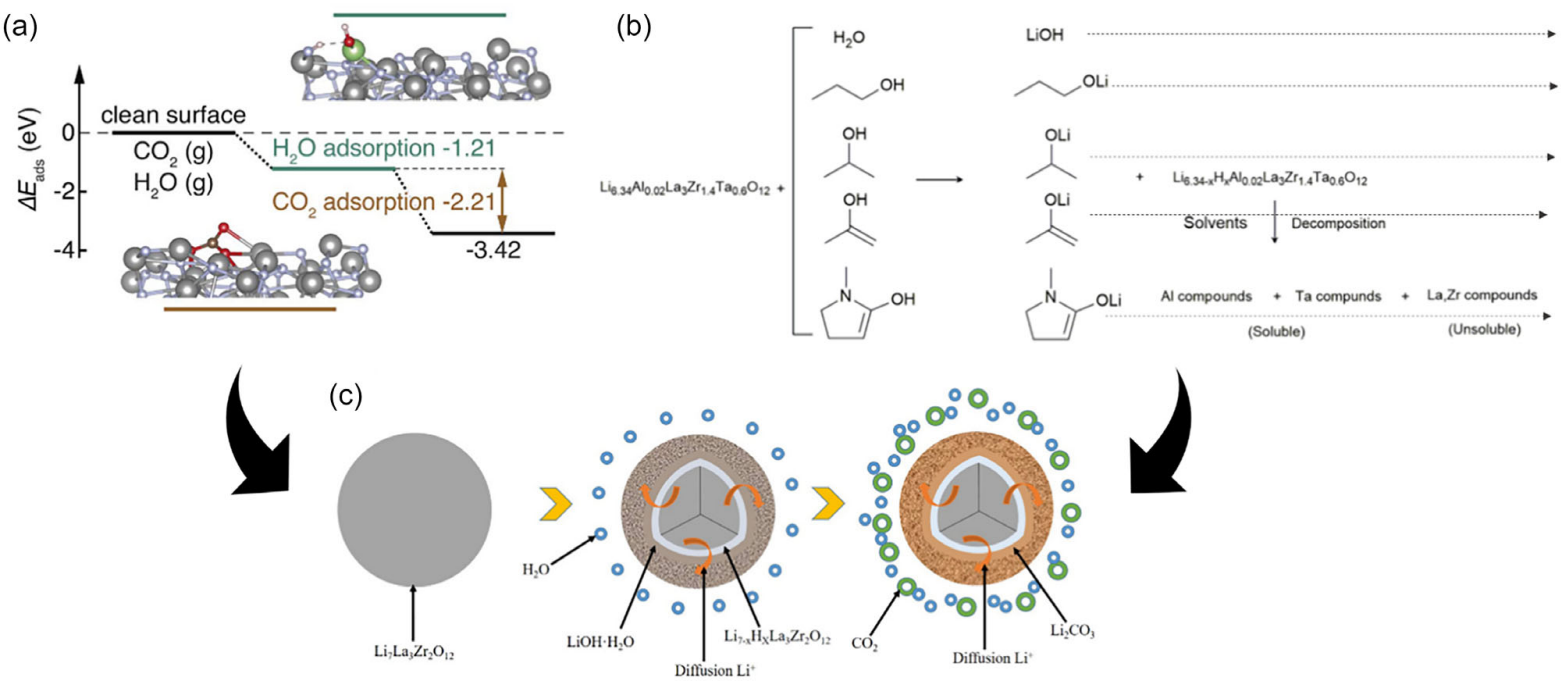
An illustration of the LLZO surface decomposition and Li_2_CO_3_ formation via protonation: (a) absorption energy diagram of protonation steps with negative calculated absorption energies, reproduced from [69] with permission. Copyright J. Mater. Chem. A (2022); (b) reaction mechanisms of LLZO protonation with different solvents, reported from [70] with permission. Copyright journal of ACS Appl. Mater. Interfaces (2021); (c) schematic illustration of the Li_2_CO_3_ formation process via protonation, reproduced from [43] with permission. Copyright J. Amer. Ceram. Soc. (2017).

Protonation of LLZO leads to the formation of LiOH and Li_2_CO_3_ on the LLZO surface when exposed to H_2_O and CO_2_ [67, 68, 69]. The most energetically favorable mechanism is the two‐step process shown in Figure 2a. When LLZO is exposed to moisture or protic solvents, Li^+^ ions are exchanged with H^+^ ions on the surface to form LiOH, which then reacts with CO_2_ from the atmosphere to form Li_2_CO_3_ on the surface of the LLZO particles (Equations (1) and (2)) [65, 71].

(1) $${\mathrm{Li}}_{7}{\mathrm{La}}_{3}{\mathrm{Zr}}_{2}O_{12}\;+\;xH_{2}O\;\to \;{\mathrm{Li}}_{7-x}H_{x}{\mathrm{La}}_{3}{\mathrm{Zr}}_{2}O_{12}\;+\;\text{x}\text{LiOH}$$

(2) $$\mathrm{LiOH}\;+\;1/2\;{\mathrm{CO}}_{2}\;\to \;1/2\;H_{2}O\;+\;1/2\;{\mathrm{Li}}_{2}{\mathrm{CO}}_{3}$$

In detail, the Li^+^ ions with the highest mobility at the *96h* site in the LLZO crystal structure tend to be replace by proton first, followed by the Li^+^ ions at the *48g* site, which are partially exchanged, while the Li^+^ ions at the *24d* site with the lowest mobility are hardly affected [72, 73, 74]. However, Ta‐ or Nb‐substitution can increase the mobility of Li^+^ at *24d* site, leading to an increased degree of protonation at the *24d* site [35, 75].

The protonation of LLZO was first observed when LLZO powders were stored in ambient atmosphere [45, 55]. Later studies by *Kun* et al. and *Grissa* et al*.* extended on these findings and showed that similar degradation occurs when LLZO comes into contact with polar and protic solvents, including water and alcohols [51, 67]. Furthermore, *K. Nie* showed that protonation can also occur in the presence of solvents with protic functional groups such as N‐methyl‐2‐pyrrolidone (NMP), which is commonly used in battery manufacturing [70]. The degradation proceeds according to the same mechanism, triggered by Li^+^/H^+^ exchange (Figure 2b). However, the degree of reaction varies from solvent to solvent because of different Brønsted acidity of hydrogen atoms in these compounds. The stronger the acidity of a solvent, the more intense the Li‐H exchange. Water and alcohol with hydroxyl groups (e.g., MeOH and EtOH) lead to stronger reactions due to their high acidity and protic nature. In all cases, the degradation leads to the formation of impurity layers, primarily Li_2_CO_3_, on the surface of LLZO particle (Figure 2c).

**FIGURE 3:**
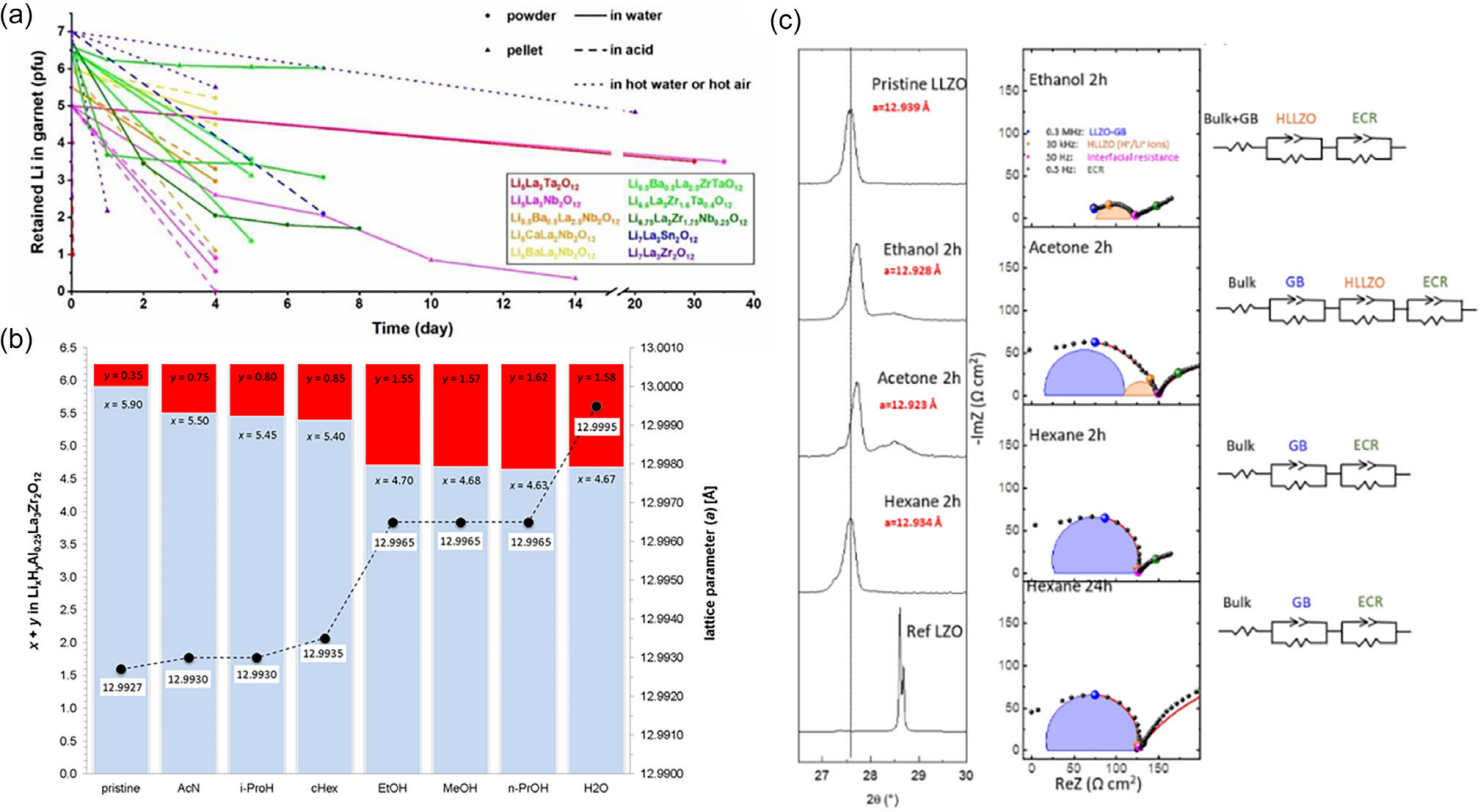
(a) Remaining Li content after treatment in different aqueous and acidic solutions for different periods. Reproduced from [77] with permission. Copyright ChemSusChem (2021); (b) synchrotron diffraction pattern for the (400) reflection of LLZO powder treated in different media. Reproduced from [51] with permission. Copyright ACS Appl. Mater. Interfaces (2018), (c) diffraction peaks of LLZO powder treated in different acidic solutions and their corresponding Nyquist plots of Li/LLZO/Li at 25°C. Reproduced from [67] with permission. Copyright ACS Appl. Mater. Interfaces (2021).

Protonation and LiOH formation increase significantly with higher temperatures, higher humidity, and longer exposure time, mainly due to faster kinetics [76, 77]. The influence of the most important environmental conditions on the reaction kinetics is summarized in Figure 3. As expected, powders often exhibit a higher degree of protonation than pellets due to their larger surface‐to‐volume ratio. In this respect, the degree of protonation for LLZO tapes should lie in between. In addition, the pH of the environment plays an important role in the kinetics of protonation. Neutral and low‐pH surroundings, for example, water and acid, promote protonation, while high‐pH environments, for example, LiOH solution, suppress protonation and can sometimes even reverse the process by relithiating the protonated garnets.^[65]^ Furthermore, the reaction kinetics is determined more by the temperature‐dependent proton diffusion within the garnet structure than by the H^+^ concentration in the environment. This indicates a significantly increased Li/H exchange rate at 90°C compared to room temperature [78]. Deep protonation leads to an increase in lattice parameters as the O—Li bond is replaced by the O–H bond (Figure 3b). In turn, impedance increases due to the formation of less conductive phases such LLZO:H, LiOH, and Li_2_CO_3_ (Figure 3c). Various dopants of LLZO (e.g., Al, Ta, Nb, and Sn) [37, 46, 68, 79, 80, 81] appear to have little or no effect on the kinetics of LLZO protonation, as no clear correlation between the type of doping and the protonation rate could be established, nor could any doping strategy successfully eliminate this degradation pathway despite extensive investigations. Structural studies have shown that proton exchange mainly affects lithium ions occupying the octahedral sites (48g and 96h) in the LLZO lattice [68], which is likely due to the higher mobility of Li ions in these sites, making them more susceptible to exchange with H^+^ ions from the surrounding medium. Since protonation has such a profound effect on the structure and stability of LLZO, it is not just a theoretical problem, but a critical factor in the formulation of the slurry for tape casting. But how exactly does this phenomenon affect the casting process and influence the stability of the slurry, the quality of the green tape, and ultimately the performance of the final components? To answer this question, in the next section we look at the practical challenges of casting LLZO tapes and examine how protonation affects the processability and performance of the final components.

### 2.2 Influence of Protonation on the Tape Casting Process

**FIGURE 4:**
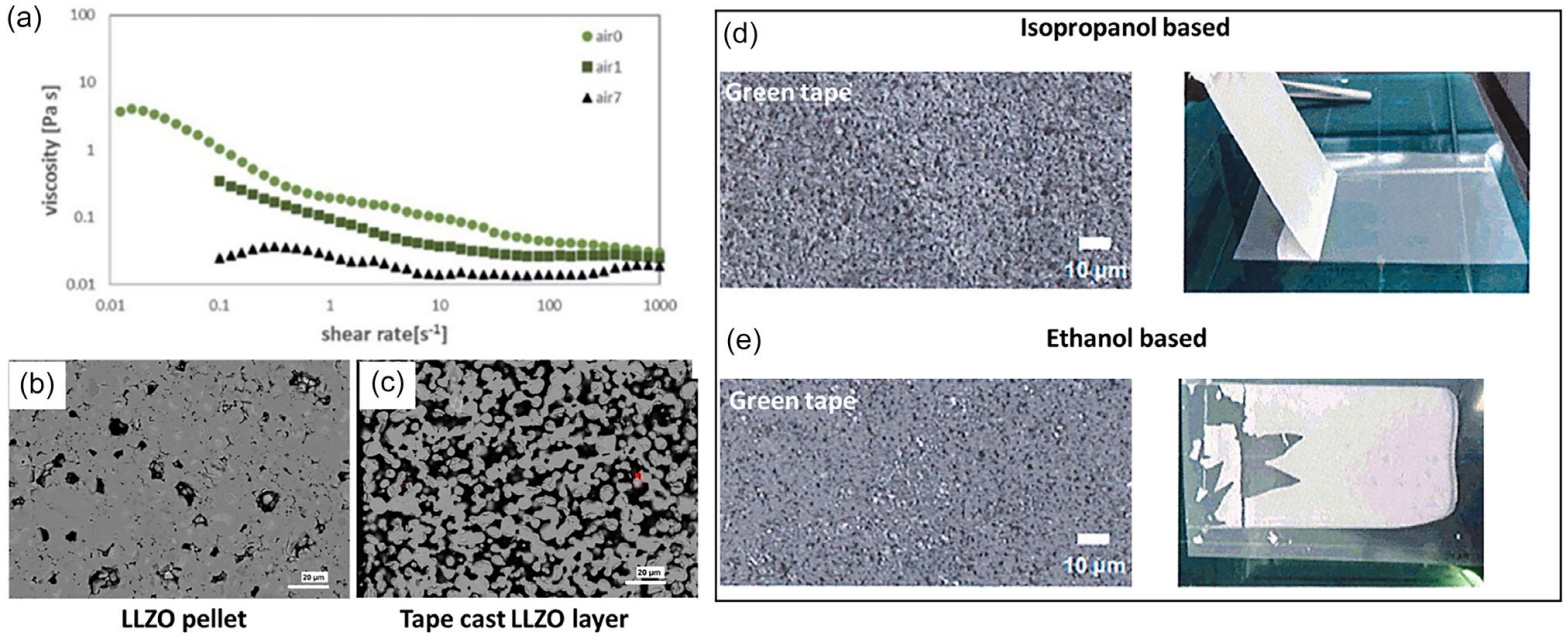
(a) Viscosity over shear rate of powders treated and stored in different atmospheres and periods (air0 = annealed in air, air1 = annealed in air and stored 1 day in air, air7 = annealed in air and stored 6 days). Reprinted from [37] with permission. Copyright J. Mater. Chem. (2021); (b,c) SEM cross section of sintered pellet and sintered tapes made of same starting powder. Reprinted from [46] with permission. Copyright Energy Storage Mater. (2024); (d,e) SEM (left) and photographs (right) of green tapes processed with different solvents (isopropanol and ethanol). Reprinted from [63] with permission. Copyright Power Source (2022). SEM indicates scanning electron microscopy.

The protonation of LLZO particles with formation of LiOH and Li_2_CO_3_ on the surface mainly occurs during the steps 1 and 2 (Figure 1) including ball milling, slurry preparation, and drying. Surface contaminants affect the wettability of LLZO particles with the binder, which in turn affects the rheology and viscosity of slurry [63, 82]. Depending on whether the LLZO surface is covered with Li_2_CO_3_, LiOH, or a combination of both, the interactions with the dispersant and binder in the slurry vary, leading to irregularities in the flow behavior of the slurry. Furthermore, both Li_2_CO_3_ and LiOH can be dissolved to a certain extent in solvents like water or ethanol. Although this interaction may not alter the particle surface as much, but the increasing pH could significantly affect the properties of the binders and lead to different rheological behavior. Such uncontrolled parameters often result in an inhomogeneous casting and, in some cases, the formation of a gel‐like structure that cannot be cast, complicating efforts to achieve a uniform, defect‐free layer. Fluctuations in environmental conditions such as temperature and humidity (especially seasonal fluctuations) further complicate the control of the protonation rate and the prediction of the type and amount of resulting Li‐containing impurities on the LLZO surface. This variability complicates the reproducibility of slurry formulations and leads to batch‐to‐batch differences. *Rosen* et al. showed that conditions and duration of storage of powders and slurry distinctly affect the viscosity profiles [37] (Figure 4a). It has also been demonstrated that the slurry properties, which are determined by the protonation state of the LLZO, have a direct influence on the properties of the sintered tapes, including morphology, porosity, and phase purity, which affect the ionic conductivity of the separator and its performance (Figure 4b,c) [83, 84]. The use of different solvents for slurry preparation also influences the mechanical properties of the dried films. For example, *A. Praejiya* et al. showed that green tapes prepared with ethanol compared to isopropanol often contain secondary phases, which is undoubtedly due to the differences in protonation rates in these solvents [63] (Figure 4d,e). In addition, the use of strongly protic solvents such as acetonitrile (AcN) can induce structural decomposition of LLZO via the Thorpe reaction, in which LLZO acts as a base catalyst to capture protons from the AcN. The results of this reaction induce polymerization, which we expect to reduce the viscosity of the slurry, and it has been shown to induce severe structural decomposition of LLZO into secondary phases such as La_2_O_2_CO_3_ and Al(OH)_3_ [70]. The presence of various protonation products, including Li_2_CO_3_, LiOH, protonated LLZO (LLZO:H), and any other possible degradation products in green tapes, complicates the sintering process, as each phase has distinct sintering temperatures and thermal shrinkage properties. As a result, nonuniform densification occurs, leading to the formation of insulating secondary phases such as La_2_Zr_2_O_7_ [50] and porous morphologies in the final tapes, as observed in the previous study [64] (Figure 4c). These issues pose significant challenges for the scalability of LLZO tape casting and the consistent production of high‐quality tapes suitable for full‐cell integration.

### 2.3 Treatment of LLZO Powders for the Fabrication of Slurries

Although several methods have been developed to remove impurities from the surface of LLZO, preventing its continuous surface degradation remains a major challenge. Coatings have been proposed as potential barriers to inhibit such surface reactions for different materials, but their practical application for LLZO is limited by several factors. In general, coatings must be applied in a dry or protonation‐limiting environment, which makes the process significantly more complex and costly. Moreover, LLZO coatings must simultaneously exhibit lithium ionic conductivity and stability at high sintering temperatures, criteria that are difficult to meet simultaneously.

To effectively control the protonation of LLZO during tape casting, it is crucial to control moisture and CO_2_ exposure. For storage, glovebox environments are a proven solution that ensure a controlled atmosphere. However, maintaining precise control over long production times during tape casting, particularly during slurry preparation and casting, poses a significant technical challenge. Several strategies have been proposed to reduce Li^+^/H^+^ exchange. One precautionary measure is to introduce excess lithium during LLZO synthesis, typically between 7 and 50 wt% [54, 57], to compensate for losses during subsequent manufacturing steps. Another widely used strategy is to heat treat LLZO at around 750°C in air. This process allows surface impurities such as Li_2_CO_3_ and LiOH to be efficiently removed from the LLZO before further processing (Equations (3) – (6)) [85, 86].

(3) $$>200{}^{\circ}C:{\mathrm{Li}}_{7-x}H_{x}{\mathrm{La}}_{3}{\mathrm{Zr}}_{2}O_{12}\cdot nH_{2}O\;\to \;{\mathrm{Li}}_{7-x}H_{x}{\mathrm{La}}_{3}{\mathrm{Zr}}_{2}O_{12}\;+\;nH_{2}O$$

(4) $$>400{}^{\circ}C:{\mathrm{Li}}_{7-x}H_{x}{\mathrm{La}}_{3}{\mathrm{Zr}}_{2}O_{12}\;\to \;{\mathrm{Li}}_{7-x}{\mathrm{La}}_{3}{\mathrm{Zr}}_{2}O_{12-x/2}\;+\;x/2H_{2}O$$

(5) $$>650{}^{\circ}C:{\mathrm{Li}}_{7-x}H_{x}{\mathrm{La}}_{3}{\mathrm{Zr}}_{2}O_{12}+x\mathrm{LiOH}\;\to \;{\mathrm{Li}}_{7}{\mathrm{La}}_{3}{\mathrm{Zr}}_{2}O_{12}\;+\;xH_{2}O$$

(6) $${\mathrm{Li}}_{7-x}{\mathrm{La}}_{3}{\mathrm{Zr}}_{2}O_{12-x/2}+x/2{\mathrm{Li}}_{2}{\mathrm{CO}}_{3}\;\to \;{\mathrm{Li}}_{7}{\mathrm{La}}_{3}{\mathrm{Zr}}_{2}O_{12}\;+\;x/2{\mathrm{CO}}_{2}$$

**FIGURE 5:**
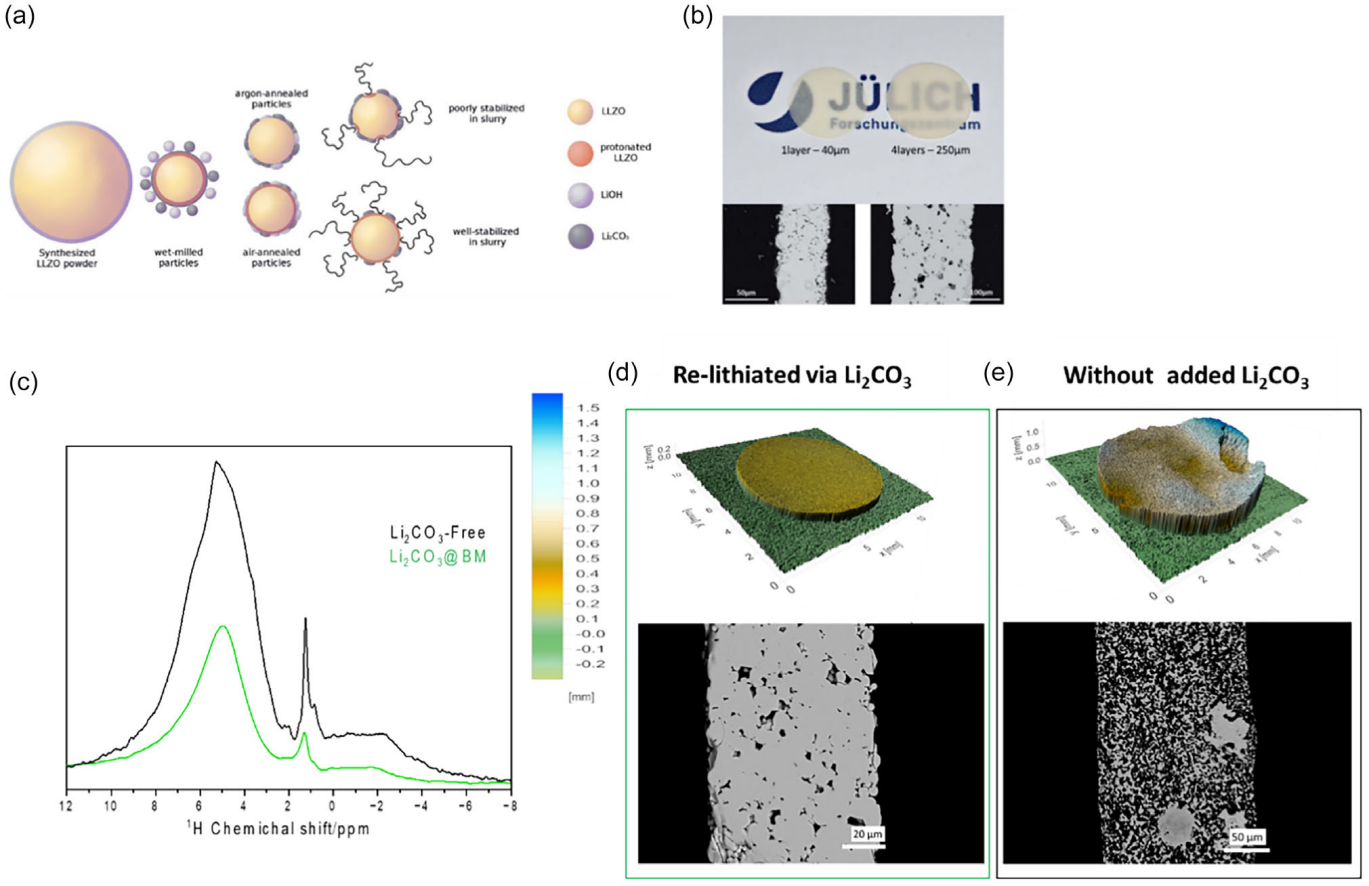
(a) Illustration of LLZO surface coverage subjected to different heat treatments and their effect on slurry stabilization; (b) the SEM and photographs of sintered LLZO tapes obtained via air heat‐treated LLZO powder. Reprinted from [37] with permission from J. Mater. Chem. (2021); (c) comparison of proton SS‐NMR of protonated LLZO powder (black) and relithiated powder by Li_2_CO_3_ (green). (d,e) C–omparison of 3D‐light topography profiles (top) and SEM cross sections of sintered tapes obtained from lithiated and nonlithiated LLZO. Reprinted from [46] with permission, copyright of Energy Storage Mater. (2024).

*Rosen* et al*.* have demonstrated the effectiveness of heat treatment in increasing the reproducibility of the tape casting process. By removing surface contaminants such as Li_2_CO_3_, the fresh LLZO surface is better exposed to the dispersants in the slurry, promoting uniform coverage and stabilization of the particles [37], as shown in Figure 5a, and resulting in a dense and stable separator (Figure 5b). To further reduce the formation of Li_2_CO_3_ during slurry preparation, a premixing step of the binder solution (consisting of binder, solvent, and additives) is introduced prior to the addition of LLZO. While dispersants are typically added before the binder to improve particle dispersion and break up agglomerates, this modified approach aims to reduce the direct contact time of the solvent with LLZO and has proven to be a helpful strategy [84]. In previous research [46], we showed that the incorporation of Li_2_CO_3_ during wet ball milling enables relithiation of LLZO by shifting the equilibrium of the protonation reaction (Equations (1) and (2)) to the left. This finding was supported by solid‐state H‐NMR analysis, which revealed a lower degree of protonation in samples milled with an additional Li_2_CO_3_, resulting in a high‐quality LLZO sintered separator, as shown in Figure 5d.

## 3 Main Problems during the Sintering Step

### 3.1 Lithium Loss at High Temperatures

**FIGURE 6:**
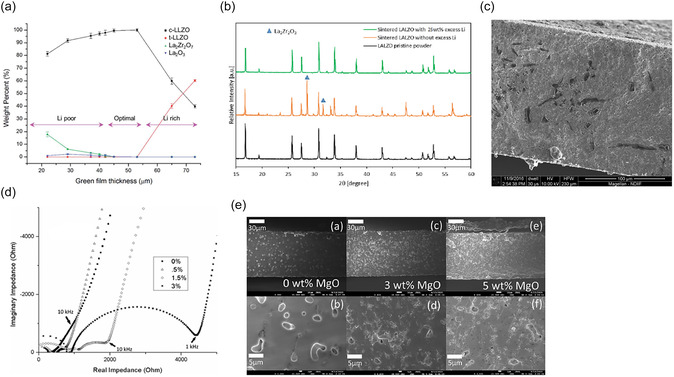
(a) Diagram of LLZO phase composition with thickness of the films heated in the same conditions. Reprinted from [57] with permission. Copyright J. Mater. Chem. A (2016); (b) XRD patterns of sintered films with and without Li excess. Reprinted from [63] with permission, Copyright J. Power Source (2022); (c) SEM image of sintered LLZO showing the segregation of Li_3_BO_3_ sintering aid; (d) EIS plots showing the effect of the amount of Li_3_BO_3_ as sintering aid on the resistance of sintered tapes. Reprinted from [54] with permission. Copyright Solis State Ionics (2018); (e) SEM images of sintered tapes showing impact of the MgO addition to the microstructure. Reprinted from [55] with permission. Copyright Energy and Fuel (2021).

Conventional sintering of LLZO requires high temperatures above 1050°C and long dwell times of typically 6–36 h [45, 61, 87, 88, 89] to achieve sufficient grain growth, neck formation, and pore elimination for dense ceramic tapes [90]. The exact sintering temperature depends on the LLZO dopant, as different dopants slightly alter the thermal behavior of a material and the onset of densification. Nevertheless, such high‐temperature treatment inevitably leads to lithium loss due to Li vitalization according to Equation (7) [91] or reactions with the sintering substrate material [45]. For this reason, virtually all reported LLZO sintering protocols use an excess of lithium precursor in the starting composition to compensate for lithium loss and surface reactions during high‐temperature processing. Lithium in LLZO can also react with atmospheric CO_2_ at temperatures as low as 700 K [66], which can lead to Li depletion during cooling. Thin LLZO tapes are particularly susceptible to lithium loss due to their high surface‐to‐volume ratio, as demonstrated by *Yi* et al*.* [57] (Figure 6a). This increased Li loss promotes the formation of Li‐poor phases such as La_2_Zr_2_O_7_ and La_2_O_2_CO_3_ according to Equation (8) [66], which reduce ionic conductivity [57].

(7) $${\mathrm{Li}}_{2}O_{\left(s\right)}\;\to \;2\mathrm{Li}\left(g\right)\;+\;1/2\;O_{2}$$

(8) $${\mathrm{Li}}_{7}{\mathrm{La}}_{3}{\mathrm{Zr}}_{2}O_{12}\;+\;4\;{\mathrm{CO}}_{2}\;\to \;7/2\;{\mathrm{Li}}_{2}{\mathrm{CO}}_{3}\;+\;1/2\;{\mathrm{La}}_{2}O_{2}{\mathrm{CO}}_{3}\;+\;{\mathrm{La}}_{2}{\mathrm{Zr}}_{2}O_{7}$$

The formation of such secondary phases is generally considered to be one of the main reasons for the deterioration of ionic conductivity in LLZO. However, the electrochemical performance of LLZO is not determined solely by its phase composition. Even for predominantly tetragonal LLZO, high ionic conductivities of up to ∼3 × 10^−4^ S cm^−1^ at room temperature have been reported, demonstrating that phase composition alone does not determine ion transport [92]. This suggests that, in addition to phase composition, microstructure is also a critical factor for ion transport in LLZO. Consequently, achieving a high degree of densification becomes a primary objective in the sintering of LLZO, as this directly influences microstructural quality and thus effective ionic conductivity. The densification of LLZO can be carried out in ambient air. However, several studies have used inert atmospheres (Ar or N_2_) or pure oxygen to suppress Li evaporation and reactions between lithium and atmospheric CO_2_ during high‐temperature sintering [54, 57, 60]. These controlled atmospheres have been shown to preserve the chemical composition and cubic structure of LLZO. In particular, an oxygen‐rich atmosphere often results in higher densities and ionic conductivities due to the high oxygen partial pressure, which inhibits the reaction described in Equation (8) [91]. However, sintering in inert gas is often performed when graphite sheets or carbon‐based setters are used as sintering supports to prevent Li escape and ensure the flatness of the films, as will be discussed later. Therefore, the inert atmosphere becomes necessary to prevent the oxidation of carbon by air and oxygen and to exclude the formation of Li_2_CO_3_.

In this regard, there is no single best sintering recipe for LLZO tapes; rather, a variety of temperature−time–atmosphere combinations has been experimentally investigated, as summarized in Table 4. The final performance of LLZO tapes cannot be attributed solely to the sintering parameters. Variations in the chemistry of LLZO dopants, the powder synthesis route (which determines crystallinity, particle size, and sintering reactivity), the cell architecture, and differences in slurry formulation and tape casting procedure all have a decisive influence on densification and ionic conductivity. Consequently, even LLZO tapes produced from identical powders and sintered under the same conditions can exhibit markedly different properties, as differences in slurry formulation and tape‐casting steps (dispersion quality, pretreatment, and surface contamination) can influence the sintering kinetics and the final microstructure, resulting in noticeable differences in density and ionic conductivity.

**TABLE 4:** Reported sintering conditions for LLZO tapes and their corresponding densities and ionic conductivities.

LLZO :structure dopant	Sintering steps	Temperature, *T* = dwelling time, °C, h	Atmosphere	Relative density of tapes after sintering, %	Conductivity of tapes after sintering, S cm^−1^	Ref
LLZO:Al (SSR)	2	(600°C, 1 h)	Ambient	‐‐	0.8·10^−4^	[53]
		1250°C, 5 h)				
LLZO:Al, Ta (SSR)	1	(1175°C, 10 h)	Ambient	92.8	3.9·10^−4^	[37]
LLZO:Al, Ta (SSR)	1	(1175°C, 10 h)	Ambient	95	1.8·10^−4^	[46]
LLZO:Nb/ Li_3_BO_3_ (SSR)	2	(650°C, 1 h)	Ambient	90.8	2.8·10^−4^	[54]
		(1000°C, 10 h)	Argon			
LLZO:Al (SSR)	3	(600°C, 12 h)	Argon	>90	__	[63]
		(1100°C, 12 h)				
		(500°C, 2 h)	Air			
LLZO:Al:MgO(commercial)	2	(700°C, 2 h)	Air	>90	4.2.10^−4^	[55]
		(1115°C, 3 h)	Argon			
LLZO:Ta (SSR)/ Li_2_O	2	(650°C, 1 h)	Air	99	5.2·10^−4^	[56]
		(1100°C, 6 h)				
LLZO:Al (LF‐FSP)	2	(1090°C, 1 h)	Nitrogen	94	2·10^−4^	[57]
		(800°C, 4 h)	Oxygen			
LLZO:Al, Ta (SSR)	2	(1175°C, 4 h) (800°C, 1 h)	Air Argon	90	1.5·10^−4^	[58]
LLZO:Al	__	__	__	95	5·10^−4^	[59]
LLZO:Nb, Ca	1	(1050°C, __h)	Oxygen	Porous bilayer	5.35·10^−4^	[60]
LLZO:Ca, Nb (SSR)	2	(700°C, 4 h)	__	Porous bilayer	2.2·10^−4^	[61, 62]
		(1100°C, __h)				
LLZO:Ta/Al_2_O_3_(SSR)	3	(350°C, 4 h)	__	Al_2_O_3_ pellet‐supported porous layer	__	[64]
		(700°C, 2 h)				
		(1140°C, 5 h)				

Abbrevaitions: LF‐FSP, liquid‐feed flame spray pyrolysis; SSR, Solid state reaction.

In practice, the use of inert gas is less economically attractive for large‐scale production, which is why many published studies resort to using air. Sealed crucibles and lithium‐rich “mother powder” are often used as technical measures to reduce the risk of Li loss and maintain a Li‐rich environment. Li loss at high temperatures leads to the formation of Li‐poor phases, manly pyrochlore La_2_Zr_2_O_7_, as can be seen from the difference in the X‐ray diffraction (XRD) pattern of tapes sintered with and without Li excess (Figure 6b). Another widely used strategy is the addition of sintering aids to decrease the sintering temperature, sintering time, or both. Al_2_O_3_ [88] in pelletized LLZO can form eutectic phases such as LiAlO_2_ during sintering, which can trap mobile lithium in grain boundaries, thereby improving densification and reducing Li loss [93]. Although these interactions have primarily been demonstrated in pellets, it can be assumed that the same chemical reaction pathways also occur in tape‐cast LLZO. Li_3_BO_3_ [54], MgO [55], Li_2_O [23], and ZnO [53] have been investigated in tape‐cast systems, with each of these materials influencing densification through different mechanisms. Li_3_BO_3_ and Li_2_O improve densification by forming a liquid phase at low temperatures, which improves particle rearrangement and reduces residual porosity, thereby increasing conductivity. However, higher Li_3_BO_3_ concentrations lead to borate‐rich intergranular phases (Figure 6c), which increase impedance (Figure 6d) at dosages greater than 1.5 wt%. ZnO acts via a similar liquid‐phase mechanism that reduces porosity, presumably by increasing capillary forces and promoting grain relocation. *Hanc* et a. [53] report that 0.05 wt% ZnO also improves sinterability and lowers the temperature of garnet formation with increased ionic conductivity. However, at higher ZnO loadings, Zn reacts with the Al‐containing support substrates used during sintering and forms secondary phases such as LiAlO_2_, LaAlO_3_, and La_2_Li_0__.__5_Al_0__.__5_O_4_. These boundary phases reduce both density and conductivity, highlighting the narrow optimal window for ZnO dosage. MgO reduces Li loss through a mechanism that acts on the microstructural evolution during sintering. Since MgO has a very high melting point [94], it does not form eutectic or liquid phases with LLZO, but remains chemically inert. During the initial and intermediate sintering stages, MgO distributes at LLZO grain boundaries and gradually segregates toward the triple grain junctions [95]. This redistribution suppresses abnormal grain growth and increases the tortuosity of the grain boundary paths. As a result, the outward flux of Li^+^ toward the surface is reduced, which indirectly hinders the long‐range Li diffusion along the grain‐boundary network and mitigates lithium volatilization during high‐temperature sintering [96]. As demonstrated by J*onson* et al*.* [55], MgO generates uniform microstructures with minimal formation of secondary phases, while an excess of MgO and prolonged sintering dwell times lead to boundary segregation, as seen in Figure 6e [55]. It should be noted that densification and ionic conductivity do not always correlate, as an increase in density can be accompanied by the formation of resistive secondary phases as segregations, as shown by the trends summarized in Table 5. These secondary phases reduce effective Li‐ion transport and lead to local fluctuations in ionic conductivity and electronic blocking, resulting in current density distribution during cycling [97]. The localizations of current constriction (or current hotspots) significantly reduce the critical current density (CCD) and promote the formation of filaments or dendrites, even when LLZO has a dense structure [98, 99].

**TABLE 5:** Summary of reported sintering additives for LLZO and their influence on densification and ionic conductivity under different sintering conditions.

Additive	LLZO:dopant	Sintering temperature, °C	Dosage, Wt%	Density, %	Ionic conductivity, S cm^−1^	Ref
Li_3_BO_3_	LLZO:Nb	1000 (6 h)	0	92.3	1.28·10^−4^	[69]
			0.5	90.8	2.82·10^−4^	
			1.5	83.0	9.08·10^−5^	
			3	85.9	7.21·10^−5^	
Li_2_O	LLZO:Ta	1100 (6 h)	__	99	5.2·10^−4^	[56]
MgO	LLZO:Al	1115 (3 h)	0	89.3	2.9·10^−4^	[55]
			3	87.8	4.2·10^−4^	
			5	91.0	2.3·10^−4^	
			7	91	0.3·10^−4^	
ZnO	LLZO:Al	1250 (5 h)	0.05	__	0.8·10^−4^	[53]

A direct comparison of sintering aids between studies remains difficult due to differences in LLZO dopants, sintering temperatures, and dwell times, all of which have a significantly impact on microstructure and transport properties.

### 3.2 Deformation of Tapes During Sintering and the Influence of the Sintering Substrate

**FIGURE 7:**
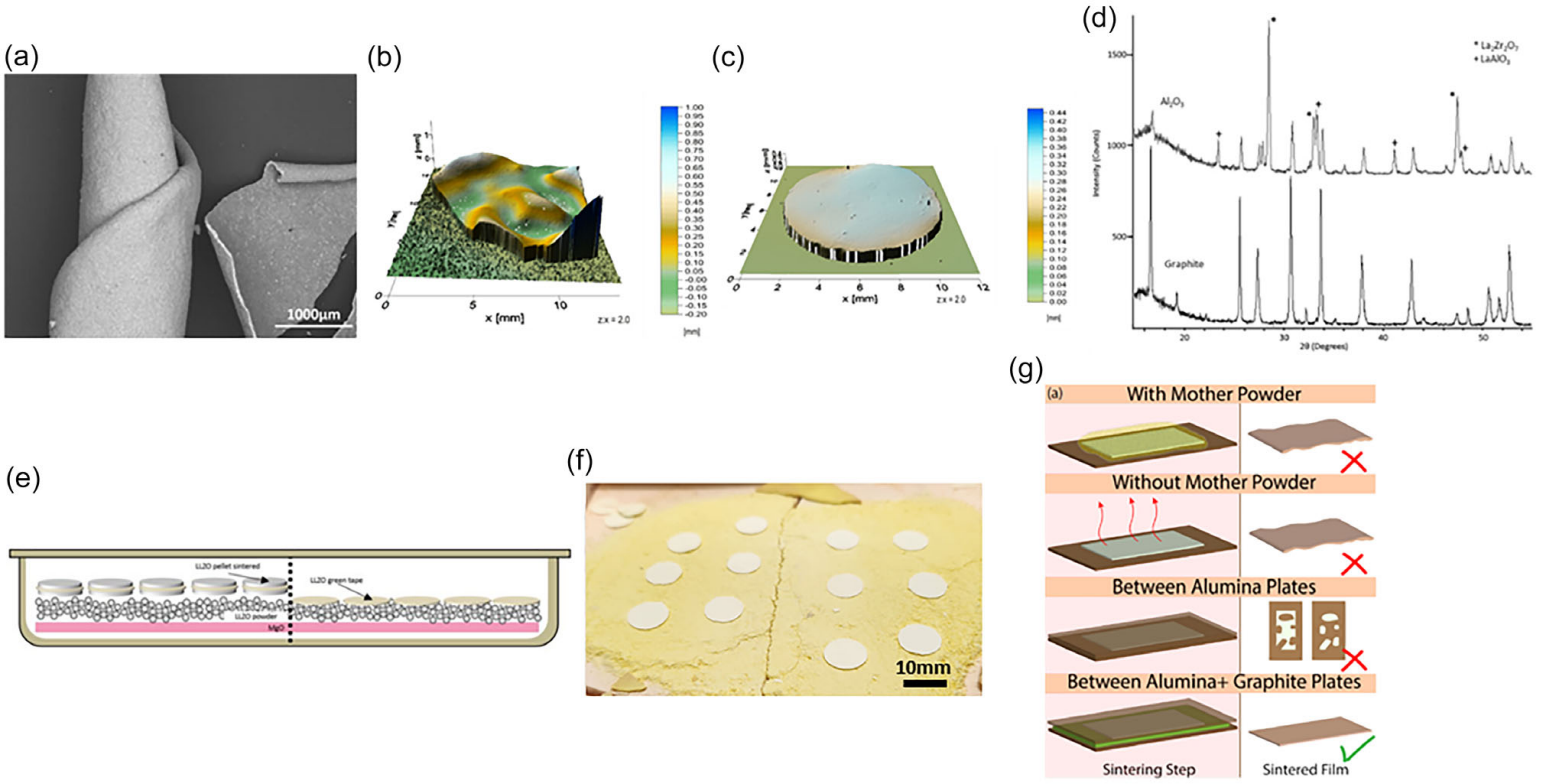
(a) SEM of sintered LLZO tape made of gradient particle size; (b,c) 3D light topography of sintered tapes with and without support. Reprinted from [84] with permission from M. Rosen; (d) XRD patterns of LLZO‐Li_3_BO_3_ tapes sintered between graphite sheets in argon (bottom) and Al_2_O_3_ plates (top). Reprinted from [54] with permission. Copyright Solid State Ionics (2018); (e) scheme representing possible sintering protocols for LLZO tapes (left) between LLZO presintered pellets and (right) by using sintering bed of LLZO mother powder; (f) photographs of LLZO sintered tapes using a mother bed protocol. (g) Diagram of approaches undertaken to carry out during the densification sintering of the green LLZO films. Reprinted from [63] with permission, Copyright J. Power Source (2022).

As LLZO tape casting continues to evolve toward scalability and industrial application, its high‐temperature sintering process presents several interrelated challenges that affect both structural integrity and long‐term performance. These challenges include adhesion to the crucible or sintering substrate, cracking, elemental diffusion, tape bending, and warping, all of which reduce production yield and raise concerns about the reproducibility and reliability of the final separator properties. One of the most persistent problems is crucible adhesion, caused by the formation of sintering necks between the LLZO tapes and the sintering substrate [100]. This causes the tapes to stick to the crucible surface after sintering [84], often resulting in mechanical damage and significant yield losses during removal. The quality of the powder bed used during sintering also influences the shape and surface quality of the tapes. An inhomogeneous, coarse, or poorly leveled powder bed can cause local height variations, resulting in a wavy surface and increased roughness of the sintered LLZO sheets. In addition, such an uneven substrate can locally restrict free shrinkage, thereby increasing the deformation of the tapes. Sheet deformation (warping, bending, and curling) is another critical defect frequently observed during sintering of LLZO films. A major cause is the nonuniform particle size distribution in the green tape, where finer LLZO particles sinter and densify earlier than coarser particles, creating internal shrinkage gradients. As shown by *Rosen* et al*.* [84], LLZO layers with pronounced particle size gradients can even roll up completely during sintering (Figure 7a). Uneven binder distribution or incomplete binder burnout can also lead to asymmetric porosity and nonuniform mass transport during sintering, resulting in deformation of the sheets. Similar effects occur when the green tape contains chemically heterogeneous regions in which secondary phases form and densify at different temperatures, leading to locally uneven sintering kinetics. These variations in densification result in spatially uneven shrinkage and the development of asymmetric internal stresses within the tape. As the tape cools, these stresses manifest themselves as macroscopic curvature after sintering [46, 100]. While these defects may appear minor on a laboratory scale, on an industrial scale they lead to significant production losses and increased material waste, which is a major obstacle to commercialization.

Another critical challenge is element cross‐diffusion at elevated temperatures. It is known that elements from the sintering support or crucible material, such as Al^3+^, can diffuse into LLZO during sintering, leading to changes in composition and structure [42]. In some cases, the diffused elements can act as sintering aids [101] and improve densification. More often, however, this leads to undesired phase transformations and a deterioration in the properties of the final product [54]. For example, Al^3+^ from alumina support plates can easily substitute Li^+^ ions in the LLZO structure, especially under conditions where lithium loss occurs at elevated temperatures. The incorporation of Al into LLZO promotes the formation of Li‐deficient secondary phases such as La_2_Zr_2_O_7_ and LaAlO_3_. This behavior was demonstrated by *Jonson* et al*.* [54], who observed the appearance of these phases in XRD patterns when LLZO green tapes were sintered on alumina plates (Figure 7d).

The above‐mentioned problems are interrelated, and several practical strategies have been implemented to mitigate them. To avoid element cross‐diffusion and to support the flatness of the sheets during sintering, LLZO films are commonly sintered on a bed of “mother powder,” typically a compacted LLZO powder or a presintered LLZO pellet. Figure 7e illustrates these methods schematically. The mother powder or pellet layer acts as both a physical and chemical barrier, preventing direct contact with the crucible and thus reducing unwanted reactions with the support material [83, 84]. It also provides a mechanically compatible substrate that helps maintain tape flatness during shrinkage. Figure 7f shows photographs of sintered LLZO tapes on a compacted mother powder bed. The improvement in tape flatness is evident when comparing LLZO films sintered on a mother powder layer with those sintered directly on a bare substrate, as shown in the 3D topography images (Figure 7b,c).

However, the mother powder or pellets degrade after repeated use and lose their ability to act as a barrier layer; therefore, they often need to be replaced with fresh material. This approach therefore has limitations in terms of scaling up for industrial production, as the large quantities of mother powder required lead to high material and operating costs. A similar strategy involves placing green tapes between graphite plates [63], which prevents element diffusion and provides structural support to prevent the tapes from bending. *Parejiya* et al. [63]. reported that graphite “sandwich” assemblies result in flatter LLZO tapes compared to those sintered on mother powder alone (Figure 7g). Although this method effectively produces flat, nonadhering tapes, it still has significant drawbacks. As can be seen in the XRD patterns of graphite‐supported tapes (Figure 7d), reflection indicative of residual graphite is clearly visible in LLZO after sintering. This suggests carbon contamination or incomplete graphite removal, requiring additional high‐temperature air treatment to remove the residual carbon. These additional steps increase manufacturing complexity, operating costs, and processing time, making the method less attractive for large‐scale applications [84].

Looking ahead, these challenges underscore the urgent need for scalable, reproducible sintering solutions that strike a balance between compaction, phase stability, and cost efficiency. While current approaches offer incremental improvements, their practical implementation at an industrial scale remains limited. The next challenge in LLZO separator manufacturing must consider not only process optimization, but also the sustainability of materials, process, robustness, and production throughput to ensure that advances in laboratory processing translate into viable and reproducible solutions for mass production of SSBs.

### 3.3 Integration of Advanced Sintering Techniques

While conventional sintering processes for LLZO face significant challenges in terms of lithium loss and densification, advanced sintering techniques that combine high temperatures with external driving forces such as pressure or electric fields could offer an alternative. Processes such as SPS, flash sintering (FS), and ultrafast high‐temperature sintering (UHS) utilize Joule heating from electric fields to achieve rapid compaction [90], potentially overcoming lithium volatilization issues while reducing overall processing time. Although these processes have been extensively researched for thick LLZO pellets [102, 103], their application to thin films is still in its early stages. A key challenge lies in the organic binders in the green tapes, which require a debinding process. Unlike bulk pellets, tape‐cast LLZO films require the organic binders to be removed prior to sintering, a step that significantly compromises mechanical integrity and makes handling the tapes after debinding very difficult. Sintering techniques that use carbon molds or foils (such as SPS or UHS) can introduce carbon contamination or cause partial reduction of LLZO, requiring additional high‐temperature air treatment [26]. Despite these challenges, these processes show promise for the rapid fabrication of LLZO thin films. Initial publications in this field report phase stability and promising electrochemical performance at current densities greater than 1 mA·cm^−2^ [39], while reproducibility and production yield are not specified.

## 4 Postprocessing of Sintered Tapes

After successful sintering, postprocessing steps of the LLZO‐based components can boost performance to meet application‐specific requirements. These treatments involve modification of surface properties to improve compatibility with electrode materials [66, 104] and good Li interface [24] and thus ensure optimal performance and integration in SSBs. The following sections discuss key postprocessing techniques, their impact on the properties of LLZO separators, and the challenges associated with scaling them for industrial production.

### 4.1 Optimization of the Surface

**FIGURE 8:**
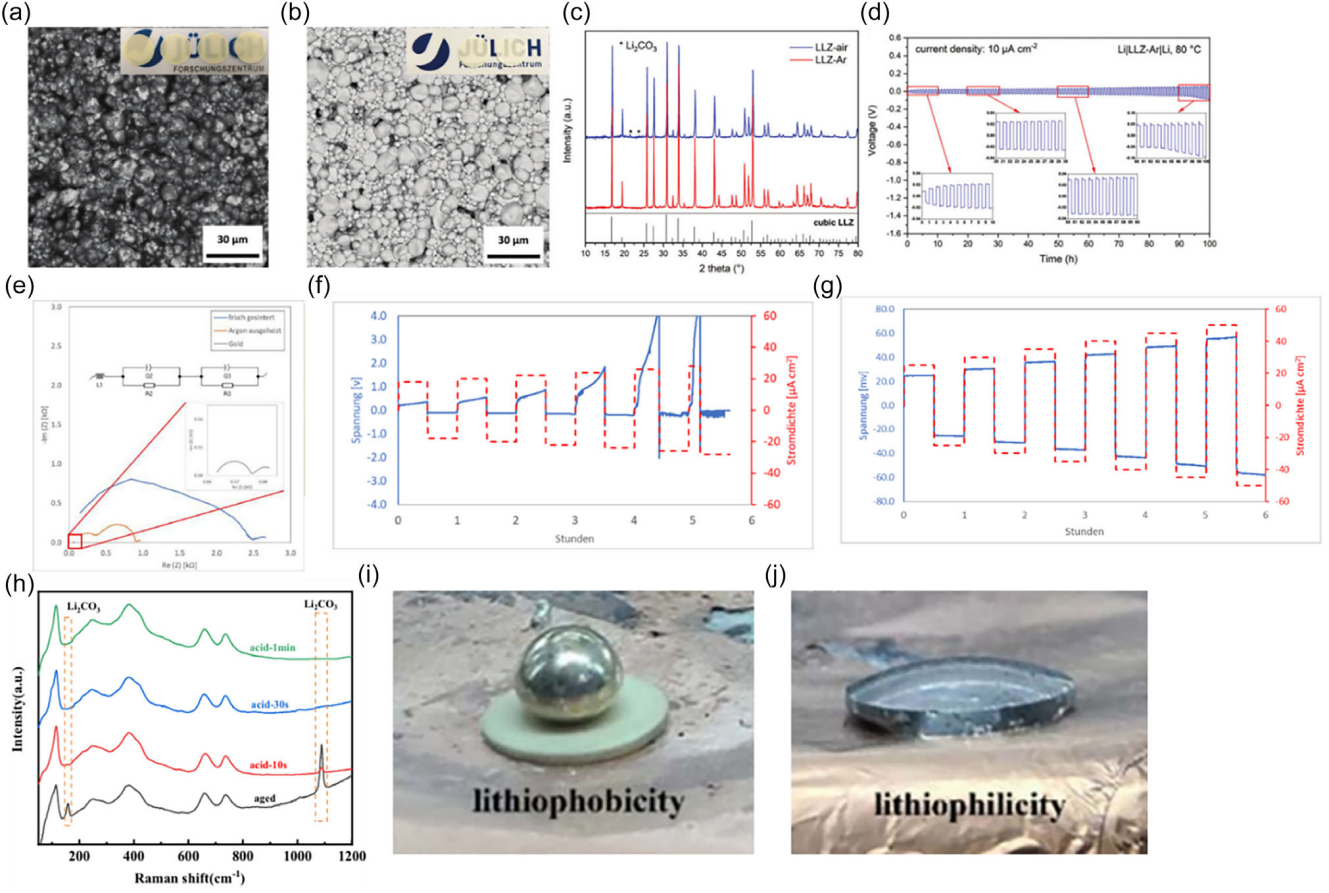
(a) Surficial SEM image of LLZ‐air (inset: photograph of LLZ‐air); (b) surficial SEM image LLZO‐Ar (inset: photograph of LLZO‐air); (c) XRD patterns of LLZO‐air and LLZO‐Ar; (d) galvanostatic cycling of a Li|LLZO‐Ar|Li symmetric cell at 10 µA cm^−2^ at 80°C. Reprinted from [58] with permission. Copyright Green Chemistry (2020); (e) impedance spectrum of symmetric Li|LLZO|Li cells of the freshly sintered LLZO films and argon baked films at room temperature; (f) CCD by DC cycling of freshly sintered LLZO samples; (g) CCD by DC cycling of argon‐sintered LLZO samples. Reprinted from [84] with permission from M. Rosen; (h) Raman spectra of LLZTO‐aged and LLZTO with different acid treatment times from 10 s and 1 min on the solid electrolytes; (i,j) Photograph of the wettability of molten lithium and LLZTO of aged pellet and after 30 s acid treatment. Reprinted from [108] with permission. Copyright Journal of Physics (2024).

After sintering, LLZO separators remain highly susceptible to surface degradation and aging due to protonation, as described in Section 2.3. In dense separators, contact with air reportedly leads to rapid formation of Li_2_CO_3_ on the surface, with prolonged exposure also leading to the formation of Li_2_CO_3_ along grain boundaries [105]. Even under controlled glove box conditions (<0.5 ppm H_2_O, <5 ppm CO_2_), thin surface layers of LiOH and Li_2_CO_3_ can form within minutes [106]. These secondary phases significantly increase the interfacial impedance in full cells and lead to inhomogeneous current distribution between the LLZO separator and the electrodes [65]. As a result, they cause uneven contact with the Li metal and localized current density, leading to the formation and propagation of dendrites [107]. Therefore, Li_2_CO_3_ must be removed before cell assembly. For pellets, mechanical polishing is a standard method for removing surface contaminants, but this approach is unsuitable for thin films due to their fragility. Alternatively, only force‐free methods such as heat treatment can be used, as this effectively decomposes Li_2_CO_3_ (see section 2.3). This technique was used for tape‐cast LLZO separator, as demonstrated by *Ye* et al*.* [58]. In their work, SEM images (Figure 8a,b) of the as‐sintered tapes showed dark surface regions indicating an accumulation of Li_2_CO_3_, which was further confirmed by XRD (Figure 8c). The as‐sintered tapes also exhibited a yellowish appearance consistent with carbonate contamination. After annealing in argon, the tapes became more transparent, the SEM surface contrast brightened (Figure 7a), and the Li_2_CO_3_ peaks disappeared from the XRD patterns, confirming the successful removal of the carbonate. The heat‐treated tapes enabled a stable cycling for over 100 h in symmetric Li/LLZO/Li cells at 10 µA cm^−2^ (Figure 8d).

A similar effect of argon annealing on interfacial quality was reported in another study [84], where the removal of the surface carbonate layer resulted in a significant reduction in interfacial resistance in Li/LLZO/Li from about 20 kΩ before annealing to about 0.5 kΩ after annealing (Figure 8e). The authors reported that prior to heat treatment, poor adhesion between molten lithium and the LLZO surface resulted in a limited true contact area. The lithium layer could be easily peeled off the separator, and the Li surface facing the LLZO appeared dark due to reactions with adsorbed surface species. This passivated layer further contributed to the high contact resistance. After heat treatment, the LLZO surface became chemically cleaner and lithium wetting was significantly improved. The molten Li adhered uniformly to the LLZO and could no longer be removed without damaging the interface, indicating a substantial increase in the effective contact area. The electrochemical response of the symmetric cell reflected this improvement. The LLZO tapes annealed under argon showed flat and symmetric voltage plateaus during plating and stripping at current densities up to 50 µA cm^−2^, consistent with homogeneous lithium dissolution and deposition. Only above approximately 60 µA cm^−2^ did a noticeable voltage asymmetry appear, indicating the onset of interfacial limitations (Figure 8f, g). This technique was used in further studies in which Li/LLZO/Li cells exhibited CCD values in the range of 0.05–1 mA cm^−2^ [37, 46, 56]. The cost‐effectiveness of argon heat treatment for large‐scale production remains questionable. Acid treatment may be another possible method for removing surface contaminants. Acids such as HF, HCl, lactic, and citric dissolve Li_2_CO_3_ and LiOH to form soluble salts (e.g., LiCl and LiF) [109]. Lactic acid (C_3_H_6_O_3_) [108] has been shown to effectively remove Li_2_CO_3_ from LLZO pellets within 30 s of the reaction (Equation (9)).

(9) $${\mathrm{Li}}_{2}{\mathrm{CO}}_{3}\;+\;2C_{3}H_{6}O_{3}\;\to \;2C_{3}H_{5}{\mathrm{LiO}}_{3}\;+\;{\mathrm{CO}}_{2}\;+\;H_{2}O$$

Raman spectroscopy (Figure 8h) confirms the removal of the Li_2_CO_3_, resulting in a clean LLZO surface with significantly improved lithium wettability, as shown in the photographs in Figure 8i,j. As a result, the treated pellets exhibited a stable cycling for over 240 h in symmetric Li/LLZO/Li cells. Although this approach has not yet been demonstrated for LLZO thin tapes, it can be assumed that the same chemical principle applies and the reaction time for removing Li_2_CO_3_ could be even shorter due to the lower thickness of the tape‐cast layers. The advantage of this process is its low cost, which makes it attractive for scalable industrial applications. However, acid treatment also poses significant challenges, such as the risk of LLZO corrosion and protonation during prolonged treatment [110]. In contrast to heat treatment, where lithium can be reincorporated into the LLZO structure, acid treatment leads to an irreversible loss of lithium [110]. Therefore, from an industrial perspective, the storage and handling of LLZO thin films must be further optimized to ensure reliable cell integration.

### 4.2 Integration of Tape‐Cast LLZO Separators into the Cells

Due to their inherent brittleness, thin LLZO ceramic separators are susceptible to breakage during battery assembly. Therefore, strategies to integrate the LLZO separators into a battery and maintain sufficient contact to other cell components both after assembly and throughout operation are essential to ensure efficient operation of SSBs. Despite the critical nature of this challenge, most publications on LLZO tape casting do not address it, and relatively few provide complete proof‐of‐concept demonstrations in symmetric or full‐cell configurations.

Similar to the approaches for the construction of symmetrical Li/LLZO/Li cells discussed in the previous chapter, full‐cell assembly often applies Li‐metal as anode material by simply melting it onto the LLZO surface [56]. Other approaches to achieve good interfacial contact while circumventing the limited mechanical stability of thin, free‐standing separators integrate metal‐oxide interlayers [111, 112, 113] as demonstrated on LLZO pellets.

Alternative methods, such applying metal interlayers by sputtering [114, 115] or coating with lithium ethylates [116], can also be used, although these techniques presents their own challenges in terms of scalability.

**FIGURE 9:**
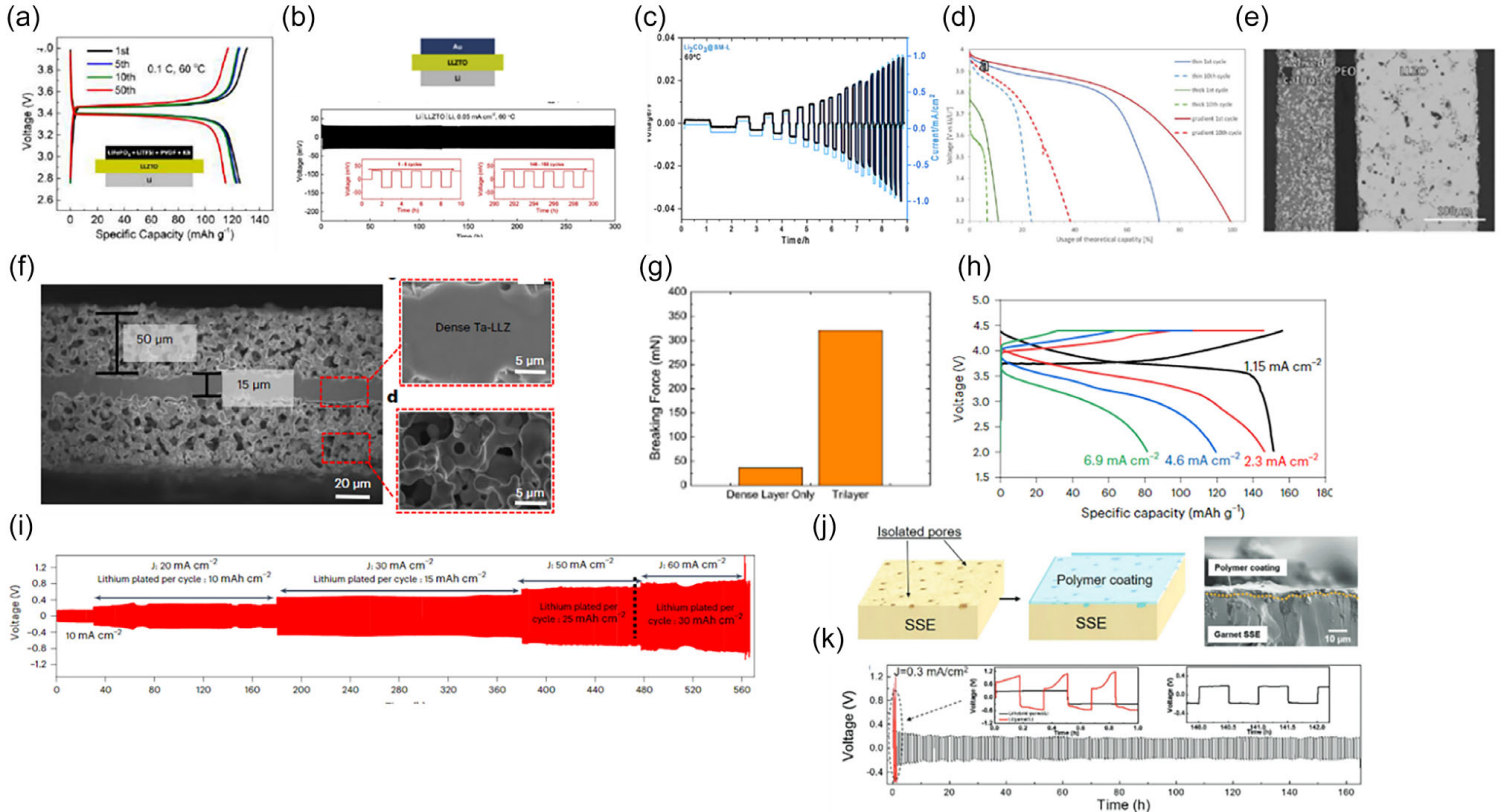
(a) Charge–discharge curves for the solid‐state Li/LLZTO/LiFePO4 battery at 0.1C; (b) voltage profiles of Li/LLZO/Au plating and striping. Reprinted from [56] with permission. Copyright J. Alloys and compounds (2019); (c) CCD of Li/LLZO/Li reaching 1mA cm^−2^ without breakage. Reprinted from [46] with permission. Copyright Energy Storage Mater. (2024); (d) discharge curves for Li/LLZO/PEO/LCO‐LLZO semi‐SSB; (e) SEM cross section of the Li/LLZO/PEO/LCO‐LLZO semi‐solid‐state battery. Reprinted from [117] with permission. Copyright J. Mater. Chem. A (2022); (f) SEM cross section of a porous–dense–porous LLZO separator. Reprinted from [118] with permission. Copyright Nat. Mater. (2023); (g) bar chart comparing the breaking force of porous/dense/porous (50/20/50 µm) separator to the dense layer only (20 µm). Reprinted from [62] with permission. Copyright Mater. Today (2029); (h) charge–discharge curves of porous/dense/porous LLZO with a Li anode and a conventional cathode (NMC622) at RT; (i) galvanostatic cycling of a symmetric Li/MIEC/LLZO/MIEC/Li cell at different current densities reaching 60 mA cm^−2^. Reprinted from [118] with permission. Copyright Nat. Mater. (2023) (j): illustration of polymer‐infiltrated porous LLZO, (k) voltage profile of the Li plating/stripping cycling at 0.3 mA cm^−2^. Reprinted from [61] with permission. Copyright Energy and Environments Science (2017).

To form a full cell, a conventional cathode slurry can be coated onto the LLZO separator [56]. This approach results in Li/LLZTO/LiFePO_4_ solid‐state cells, which have shown stable cycling at 60°C and 0.1C, delivering 125.8 mAh g^−1^ initially and retaining 92.3% of this capacity after 50 cycles (Figure 9a) [99]. In symmetric Li/LLZTO/Li cells, tape‐cast LLZO has also demonstrated stable plating‐stripping for more than 300 h at 0.05 mA cm^−2^ at 60°C (Figure 9b). Unfortunately, the total active material loading has not been reported, which prevents an evaluation of the industrial applicability.

A hybridization approach combines thin LLZO separators with free‐standing ceramic cathodes, which have also been prepared via tape‐casting, by introducing poly (ethylene oxide) (PEO) polymer as an interlayer to improve adhesion and ensure sufficient ionic conductivity at the cathode–separator interface (Figure 9e) [117]. While these cells show very high total capacities of 2.8 mAh, the rigid interfaces in the ceramic cathode promote fast capacity degradation [119].

A more promising direction is trilayer LLZO architecture with porous–dense–porous multilayer structure (Figure 9f), where the porous outer layers act as 3D ionic conductors capable of being infiltrated with cathode material. This architecture represents a significant advantage of tape casting, as the process enables the controlled fabrication of multilayer structures with tailored porosity within a single ceramic separator. Such designs were first demonstrated by the Wachsman group [61, 62, 118] and subsequently adopted and further developed by other research groups [59, 120].

Furthermore, trilayer LLZO separators exhibit up to nine times higher breaking strength than monolithic dense film separators [62], as demonstrated by three‐point bending measurements (Figure 9g). The improved mechanical properties offer significant advantages for industrial handling and assembly. In symmetric configurations, the trilayer architecture enables stable high‐current lithium cycling at room temperature when the Li|LLZO interface is appropriately engineered by introducing metal oxide interlayers that enhance lithiophilicity.

Room‐temperature galvanostatic cycling with the areal current densities of up to 10 mA cm^−2^ has been demonstrated in Li|LLZO|Li symmetric cells over extended periods of time [118]. The porous outer layers of the trilayer design increase the contact area between the electrolyte and the electrode by more than 40 times compared to traditional planar interfaces. This significantly reduces the interfacial impedance and allows higher current densities without the risk of dendrite formation.

In addition, polymer interlayers can also be infiltrated into the porous LLZO prior to lithium application [28, 61] to homogenize the surface by filling the pores and suppress dendrite‐forming hotspots (Figure 9j). Symmetric cells assembled in this manner have exhibited stable cycling for 180 h at 0.3 mA cm^−2^ (Figure 8k) [62]. This suggests that the anode side is no longer the primary bottleneck for LLZO‐based SSBs, shifting the focus to the development of cathode composites and the scalable production of separators. When combined with conventional NMC622 cathodes containing liquid electrolyte, hybrid cells incorporating trilayer LLZO demonstrated long‐term cycling stability (500 cycles at 2.3 mA cm^−2^ or 350 cycles at 1.15 mA cm^−2^ at room temperature and have also been shown to operate effectively across a range of cycling although the delivered capacity decreased (Figure 9h) [118].

Despite these promising advances in integrating LLZO tapes into full solid‐state cells, it is important to note that the most significant results so far have been achieved with hybrid cell configurations that incorporate polymers in the cathode or as interlayers. While these hybrid systems offer considerable advantages in terms of cycling stability, particularly with regard to the integration of lithium metal anodes, their performance is typically optimized for moderate operating temperatures (<100°C) due to the thermal limitations of the polymers used [121]. However, this does not diminish their industrial relevance, as these hybrid cells have demonstrated increased current densities and long‐term cycling stability under controlled conditions, making them suitable for practical applications. Notably, lithium metal can be used in these configurations, offering a nonflammable and nontoxic alternative to liquid electrolytes. While these cells show significant advances in performance, the biggest challenge now is to increase the area of these systems to achieve practical cell sizes. Recent advances in scaling LLZO‐based SSBs have successfully demonstrated the feasibility of large‐area cells with remarkable performance. In one study, a 30 cm^2^ (4.5 × 8.0 cm^2^) LLZO solid electrolyte with a 3.2 mAh cm^−2^ LiCoO_2_ cathode was achieved, which could be cycled stably for over 400 cycles at a rate of 0.5 C while retaining a capacity of about 95 mAh [122]. This represents a significant step forward in overcoming the scalability issues of LLZO‐based cells and demonstrates their potential for practical, large‐scale applications. These results underscore that the problem of achieving high current densities and long‐term cycling stability is becoming less important, and the main challenge is now shifting to optimizing production techniques for industrial‐scale deployment.

While significant progress has been made in integrating LLZO as a separator in hybrid SSBs, the realization of fully solid‐state systems requires the successful integration of LLZO with an inorganic cathode. This concept can be realized by co‐sintering LLZO with a ceramic cathode tape or co‐synthesizing cathode active material into a porous LLZO framework. Nevertheless, those concepts face fabrication challenges as co‐sintering LLZO with cathode active materials poses material compatibility during sintering. Problems such as elemental diffusion, elemental segregation, reactivity with formation of electrochemically inactive reaction products, and resistive interfaces are known to occur with LLZO [26, 123, 124, 125, 126, 127, 128, 129, 130, 131] and are even more pronounced in thin‐film architectures, where higher surface reactivity and lithium loss accelerate material degradation. These effects could significantly impair electrochemical performance and long‐term stability, making co‐sintering a complex but important area for further research.

## 5 Conclusion and Perspective

Integration of the tape casting into the fabrication of LLZO separators has made significant progress in recent years. Several studies have demonstrated the feasibility of producing thin and dense solid electrolytes that can successfully be integrated into full cells. However, the current tape‐casting workflow has several inherent bottlenecks. LLZO degradation begins at the earliest stages of processing through protonation and continues during high‐temperature sintering through Li evaporation. Degradation in one stage affects subsequent steps. We have shown how protonation during slurry preparation alters the rheology and stability of the slurry, which in turn affects the flexibility of green tape, sintering behavior, and compatibility with downstream integration steps. These challenges ultimately lead to structural changes that subsequently affect wettability with lithium, interfacial stability, and cell performance.

In this perspective article, we have highlighted these challenges step by step and, for the first time, systematically outlined how degradation at one stage affects the next. For example, Li‐H exchange during slurry preparation not only destabilizes the slurry but also introduces secondary phases that compromise the mechanical integrity of green tapes. During sintering, Li loss further increases structural inhomogeneity, and thin LLZO tapes continue to be particularly prone to sticking to the crucible and undergoing undesirable phase changes. These interdependent effects illustrate why LLZO tape casting still presents significant difficulties for industrial adoption. We have also summarized existing mitigation strategies, including the use of sintering aids, mother powders, and controlled sintering atmospheres, which help suppress Li loss and promote densification. However, these solutions are still largely in the laboratory stage and often rely on delicate processing conditions that are difficult to scale economically.

Based on the mechanistic challenges outlined in this review, several specific research directions for the future emerge:

1. **Control degradation in the earliest stages of manufacturing**: Since protonation and Li loss begin during slurry preparation, research should prioritize suppressing these processes before they accumulate in subsequent steps. Promising directions include exploring additional nonprotic or weakly protic solvents that are compatible with LLZO, developing humidity‐controlled tape‐casting environments, and implementing in situ lithiation strategies along the production line to compensate for Li deficiency from the outset.
2. **Develop cost‐effective and time‐efficient sintering strategies**: Conventional LLZO sintering requires long dwell times and high temperatures, which increase cost and promote Li evaporation. Future work should focus on implementing fast and low‐temperature‐based sintering processes, such as rapid thermal processing and FS, to improve efficiency and energy savings. Importantly, these techniques should be optimized for mass sintering of tapes, rather than the sample‐by‐sample processing.
3. **Reexamine the entire tape‐casting workflow in terms of time efficiency and stability**: The pretreatment of LLZO powder (e.g., particle size reduction) and the drying of the tapes currently involve long processing times, with overnight drying being common practice to remove the solvent before sintering. This increases the risk of degradation due to prolonged exposure to atmosphere and raises production cost. Future research should investigate the minimum time required for each step and redesign the workflow accordingly. Research into rapid drying technologies or solvent combinations that evaporate uniformly and quickly could significantly reduce Li loss and improve the quality of green tapes. Generally, LLZO is synthesized separately at high temperature before casting. Future work could explore the possibility of directly casting precursor mixtures, thereby eliminating the need for a complete thermal cycle, which would significantly reduce costs.
4. **Improve cell integration strategies**: Current approaches rely on polymer interlayers or adhesion layers to attach electrodes to LLZO, but these increase costs and introduce more interfaces with potentially lower stability and conductivity. A better long‐term sustainable solution could be co‐sintering of LLZO tapes with cathode tapes, provided that the parallel improvement of faster, low‐temperature sintering process can prevent the interfacial reactions that occur when sintering LLZO and cathode active material. The porous LLZO layers could be used to directly accommodate cathode active material synthesis, allowing for simplified processing and improved interfacial contact.
5. **Explore dry‐casting routes as a potential breakthrough technology**: Dry tape casting is an approach that is already being considered industrially for solvent‐free electrode manufacturing. For LLZO, this approach would drastically reduce exposure to moisture and CO_2_, shorten fabrication time, reduce cost, and significantly suppress protonation and Li loss. The next line of research should focus on the feasibility of adapting dry processing casting of LLZO. Dry casting of LLZO requires the establishment of conditions under which the LLZO powder can form cohesive, defect‐free green tapes without the rheological control normally provided by solvent‐based slurries. Future research in this direction should therefore investigate the properties of LLZO powder (particle size distribution, surface chemistry, and morphology) and identify suitable green binders that provide sufficient plasticity and mechanical integrity without solvents.

From an industrial perspective, the successful implementation of LLZO tape casting will depend on minimizing time‐ and atmosphere‐sensitive steps through workflow automation and integrating inline monitoring to reduce batch‐to‐batch variability. The slurry formulation should adopt nontoxic or nonflammable solvents wherever possible, and the number of additives could be minimized to improve safety and reproducibility in large‐scale production.

Finally, to reliably integrate the LLZO separator into a full cell, industrial production could benefit from automated robots and machining tools that apply the cathode layer directly after LLZO sintering or co‐process it in a single manufacturing line.

## Author Contributions

**Kaouther Touidjine**: conceptualization (lead), writing – original draft (lead), writing review & editing (equal). **Ruijie Ye**: writing – original draft (supporting), writing – review & editing (equal). **Melanie Finsterbusch‐Rosen**: conceptualization (equal), supervision (supporting), writing – review & editing (equal). **Artur Lang**: writing – review & editing (equal). **Martin Finsterbusch**: resources (lead), supervision (supporting), writing – review & editing (equal). **Dina Fattakhova‐Rohlfing**: funding acquisition (lead), supervision (lead), writing review & editing (lead).

## Acknowledgments

As a part of the DESTINY European doctorate program, the authors acknowledge funding from the European Union's Horizon2020 research and innovation program under the Marie Skłodowska‐Curie Actions COFUND grant agreement no 945357 and the Chairman Prof. Christian Masquelier. Financial support by the German Federal Ministry of Research, Technology and Space (BMFTR) as part of the clusters of competencies Festbatt 2 (projects 13XP0434A and 13XP0432B) and CatSE2 (project 13XP0510A) is gratefully acknowledged.

## Funding

This study was supported by Bundesministerium für Forschung, Technologie und Raumfahrt (BMFTR) (CatSE2:13XP0510A, Festbatt2‐Oxid: 13XP0434A and Festbatt2‐Produktion: 13XP0432B) and H2020 Marie Skłodowska‐Curie Actions (945357).

## Conflicts of Interest

The authors declare no conflicts of interest.

## Data Availability

The data that support the findings of this study are available from the corresponding author upon reasonable request.
